# Nanoparticle-Polymer Surface Functionalizations for Capacitive Energy Storage: Experimental Comparison to First Principles Simulations

**DOI:** 10.3390/ijms241713321

**Published:** 2023-08-28

**Authors:** Joshua Shipman, Binod Subedi, Christopher Keller, Brian Riggs, Scott Grayson, Douglas Chrisey

**Affiliations:** 1 Department of Physics & Engineering Physics, Tulane University, New Orleans, LA 70118, USAdchrisey@tulane.edu (D.C.); 2 Department of Chemistry, Tulane University, New Orleans, LA 70118, USA

**Keywords:** dielectric, energy storage, nanocomposites, capacitor

## Abstract

Dielectric capacitors present many advantages for large-scale energy storage, but they presently require higher energy density. We demonstrate novel high energy density polymer-nanoparticle composite capacitors utilizing thiol-ene click chemistry surface groups to bond the nanoparticles covalently to the polymer matrix. Interfacial effects in composites cannot be observed directly, and in our previous work, we examined the nanoparticle–polymer interface in silico. In this work, we experimentally examine the five surface functionalizations modeled previously, fabricating high energy density thin film capacitors to test our predictions. Results from this study, in conjunction with properties previously determined in silico, further improve the understanding of the role of surface functionalizations in composites prepared using click chemistry. The coating density of the surface functionalizations is shown to be a key factor in relating our computational results to experimental results. We show how using both coating density and our previous modeling in combination allows for prescreening of surface functionalizations for future composites, reducing experimental cost. We also demonstrate high energy density capacitors with ~20 J/cm^3^.

## 1. Introduction

Renewable energy sources are becoming ever more prevalent, but their output is typically intermittent [1]. Green energy solutions are clearly dependent on large-scale energy storage to match supply and demand. However, energy storage methods must be economical at the grid scale to be widely adopted. Conventional large-scale energy storage technologies require large amounts of space or are prohibitively expensive [2]. With their low cost, high cycle lifetime, and high power density, dielectric capacitors could be the solution for grid-scale energy storage if their energy storage capacity could be increased [3]. Many energy storage methods exist, but few are commercially viable for large-scale applications. Lithium-ion batteries are the most prominent energy storage method, but they present several downsides, such as their high cost, limited lifetime, and their precursor chemicals’ impact on the environment [4]. Pumped hydropower and compressed air are grid-scale energy storage methods that are both currently in commercial operation and compatible with intermittent power generation, but they necessitate special geographical features for water to flow from a storage height to a holding area or sealed caverns, respectively [5]. New renewable energy sources are now being constructed not solely in remote areas but in the built environment as well (e.g., rooftop solar panels), and thus, many generation locations may not be compatible with pumped hydropower’s extensive land needs. Other technologies, such as supercapacitors, require multiple advances in technology and manufacturing to reach the energy storage capacity and scale needed to make them viable [6,7], in addition to their fundamental disadvantage of not being modular solid-state devices [8]. Large-scale production of dielectric capacitors is also currently underway [9]. Polymer nanocomposites are in use in several different industries as well [10].

Dielectric capacitors are widely used in microelectronics but have been largely neglected for energy storage because of their low energy density. To remedy this, many have tried to create higher energy density materials through new material discovery [11,12,13] or by forming composites from different combinations of existing materials [14,15,16]. The two parameters that determine a capacitor’s energy storage capacity are its permittivity, also known as the dielectric constant, $\varepsilon_{r}$, and its breakdown voltage, $E_{B}$. *E_D_* is the overall energy density and $\varepsilon_{0}$ is the permittivity of free space, a constant.

(1) $$E_{D}=\frac{1}{2}\varepsilon_{0}\varepsilon_{r}E_{B}^{2}$$

Permittivity is a measure of the material’s ability to be polarized by an electric field. Breakdown voltage is the voltage at which the capacitor fails when a current is formed, and it is no longer an insulator. An increase in breakdown voltage increases the energy density quadratically compared to a linear increase by the permittivity. Many high permittivity materials, typically ceramics, have been discovered, but all have relatively low breakdown voltage, and techniques to remove extrinsic defects that are the cause of this low breakdown have proven too expensive for mass production. Conversely, many high-breakdown materials have been discovered, typically polymers, but attempts to increase their permittivity have proven to be unsuccessful, often resulting in high-loss materials.

Combining low-cost and high-breakdown polymers with high-permittivity fillers is an obvious solution to attain high energy density capacitors with low-cost manufacturing. In past research, these composites have resulted in a lower breakdown voltage [17,18] than neat polymers because the high-permittivity fillers typically introduce field-susceptible (or interfacial) defects to the high breakdown strength polymer, which derives its properties from its high uniformity. Filler geometry was thought to affect breakdown by concentrating the electric field, and various fillers have been tried to observe these interfacial effects, such as cubes, spheres, and rods, but they all have similar performance [19].

To remedy the effect of creating inhomogeneities by adding nanoparticle fillers in a crosslinked polymer matrix, adding fillers with surface functionalizations that reduce interfacial effects on the neat polymer matrix has been attempted. Brushes and other surface functionalizations have been shown to help with this problem somewhat by reducing interfacial differences [20,21,22]. High crosslinking films have not only reduced the interfacial defect effect, but they have also increased the ultimate breakdown of composite films dramatically. Specifically, using thiol-ene click chemistry to create a physical bond from the filler to the polymer host [23,24] was shown to reduce breakdown occurrence in the overall film, presumably because the covalent bond at the interface reduces the ability of electrons to gain energy. This energy increase is what leads to the ultimate destruction of the capacitor (as discussed above).

However, we have shown in our previous work that an electrical interface still exists between the nanoparticle and polymer host due to the large electric field at the interface with a different dielectric constant material. This is due to the large polarizability of the nanoparticle, especially as compared with that of the polymer matrix, and can be seen most dramatically in the case of metals (effectively infinite dielectrics), where an even greater effect from charge concentration is observed at the interface proportional to the applied field [25].

**Figure 1:**
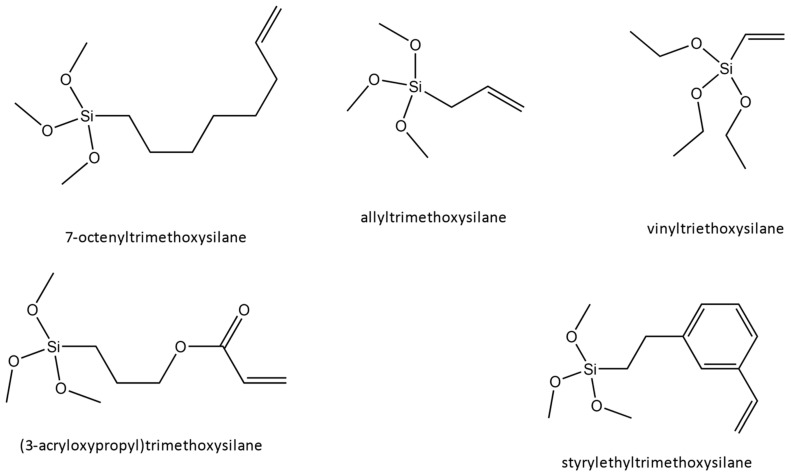
Surface functionalizations modeled in our previous work and experimentally tested in our current work, whose goal is to reduce interfacial defect effects.

In our previous work [26], we made predictions about what the highest breakdown material would be among five surface functionalizations (originally chosen for study because of chemical traits traditionally associated with high breakdown). Those surface functionalizations are shown in Figure 1, and the abbreviations used in this work are in Table 1. We performed first-principles modeling of the surface’s electric field and used this information in combination with mesoscale breakdown theory as an indicator for high breakdown. Our original prediction was that ALLY (followed by VINYL, STYR, 3ARC, and 7OCT), having the highest cumulative drop in the electric field, would lead to films with the highest breakdown. From that past work, there was no indication that any specific chemical structure was associated with breakdown in the composite (contrasting with bulk, where they are important), either by looking at the total field drop of functionalizations with similar chemical features or from trying to quantify the effect that individual features have on the electric field graph. Instead, we proposed that our method of calculating the total field drop at the interface could be used as a metric to determine which surface functionalization would create the highest breakdown composite films based on the energy gained at the interface for each electron and their corresponding effect on breakdown (elucidated through the von Hippel energy condition). In this work, we create films to test those predictions and use the resulting information to refine our model.

## 2. Results and Discussion

**Figure 2:**
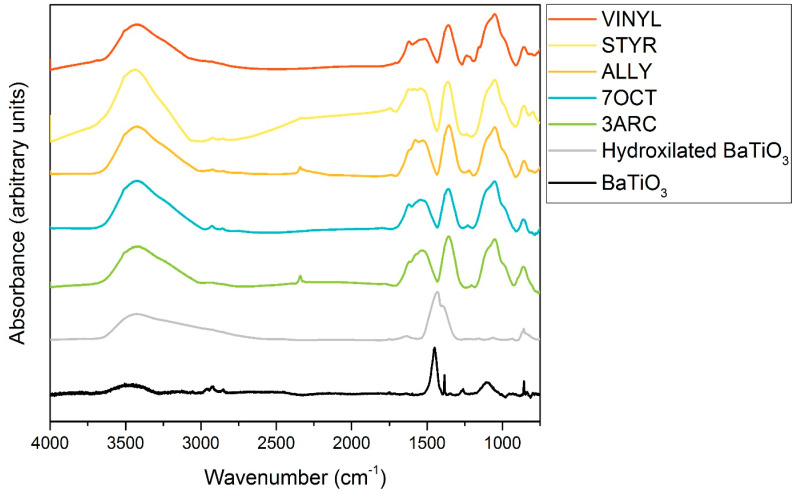

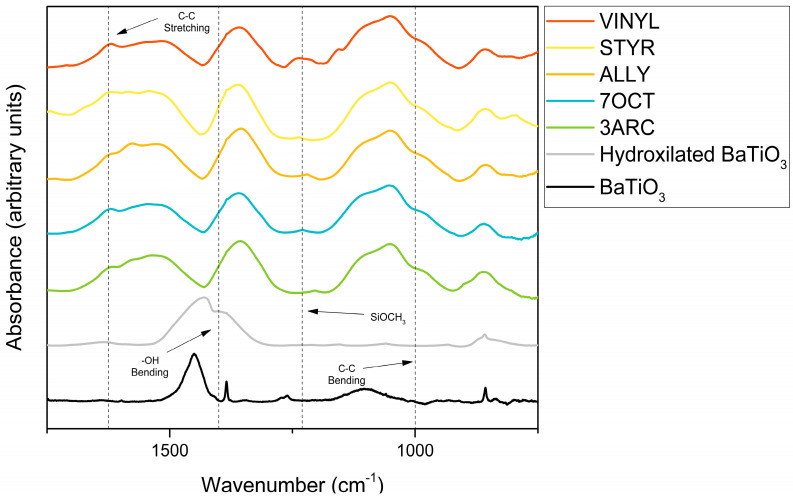
FTIR data showing bare nanoparticles, nanoparticles hydroxylated by hydrogen peroxide, and surface functionalizations used. The fingerprint region and relevant peaks are marked in the bottom graph.

**Figure 3:**
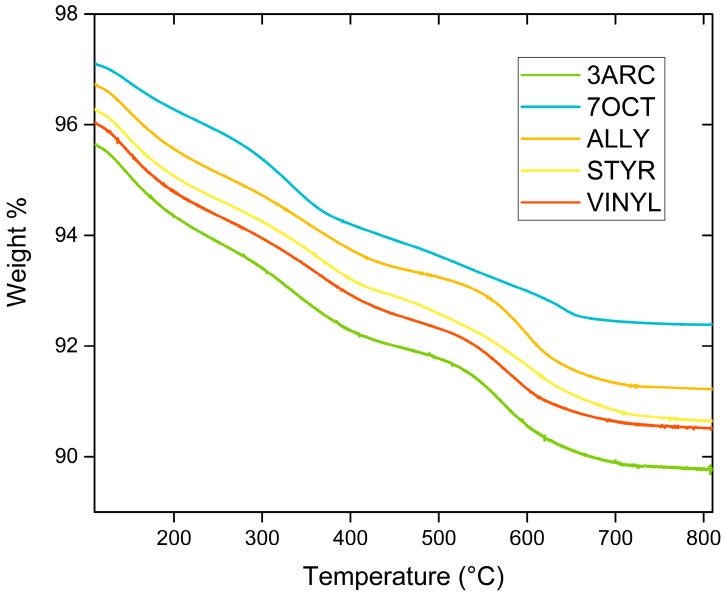
Thermogravimetric analysis of different surface functionalizations varies between 4.71 and 5.88%.

The results from FTIR spectroscopy of functionalized nanoparticles are shown in Figure 2. First, 13 mm KBr pellets with 2% weight of functionalized sample were pressed at 10 MPa for FTIR testing using a Nexus 670 FTIR ESP (Thermo Scientific, Waltham, MA, USA). FTIR was primarily conducted to confirm the surface attachment of the functionalizations. This was successfully indicated by the presence of the peaks associated with alkenes: the C-C bending peaks at around 970 and 1000 cm^−1^ and the C-C stretching peaks at around 1625 cm^−1^ in all functionalized nanoparticles. The peaks [27] centered around 1450 cm^−1^ can be attributed to residual organics left on the surface. After treatment with hydrogen peroxide, the peak, typically around 1330–1420 cm^−1^, attributed to OH bending, appears, indicating the presence of -OH groups successfully placed on the surface. The OH peak present around 3000–3500 cm^−1^ is associated with water absorbed by the pellet in processing. C-H stretching is observed for some of the compounds around 2900 cm^−1^. It might be present only on some nanoparticle surfaces because of their more extended nature, especially when compared to the most compact functionalization, ALLY. Peaks associated with aromatic rings, which could be used to positively identify STYR, as only it has aromatic rings, are typically weak and overlap with C-C stretching. The SiOCH_3_ peak at 1230 cm^−1^ is present in all samples, as expected. Figure 3 shows TGA results for the five different surface functionalizations. The weight drop among the functionalizations varies between 4.71 and 5.88%. This indicates that the films’ overall compositions are quite similar. This shows that none of the nanoparticles had enough surface functionalization to make a difference in the amount of BaTiO_3_ in each film since the weight percentage added (6%) was based on the combined weight of the nanoparticle and attached surface functionalization. This is important as we are interested in comparing the effects of the different surface functionalizations on the nanoparticle–polymer interface, and films with a large difference in the number of such interfaces would not be easy to compare. Additionally, this is not as small of a difference amongst functionalizations as it appears because of the difference in molecular weights of each functionalization.

**Figure 4:**
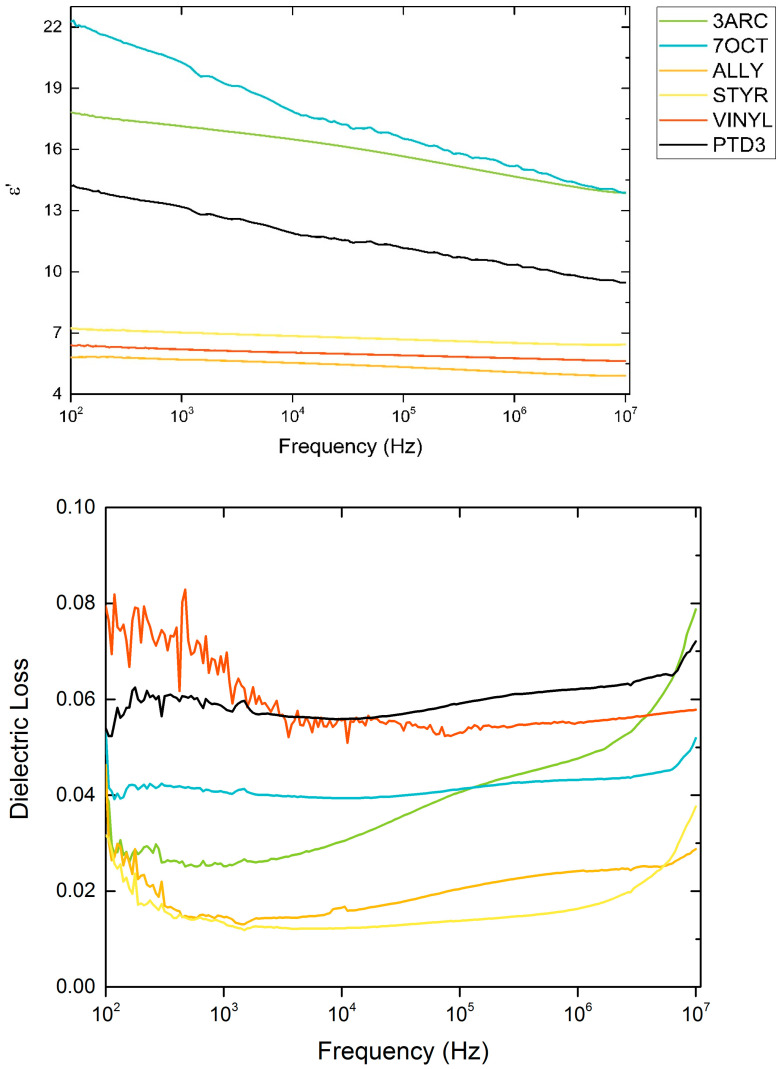
Relative permittivity and loss for films made with various functionalized nanoparticles.

Dielectric constants and loss percentages are shown in Figure 4, showing differing dielectric performance amongst the surface functionalizations. All films show losses below 10%, which is consistent with single-layer nanocomposite film capacitors. These are single-layer thin films; roll-to-roll printed MLCC-type devices should exhibit lower loss. Three films made with different surface functionalizations resulted in lower permittivity compared to neat polymer. Past research has seen this effect, which originates from the local interface created between the nanoparticle and the polymer host [28]. Lower crosslinking could also be a factor due to the spatial way that the surface functionalizations were attached to the surface, leading to spatial orientations of the surface functionalizations with low polarizability.

**Figure 5:**
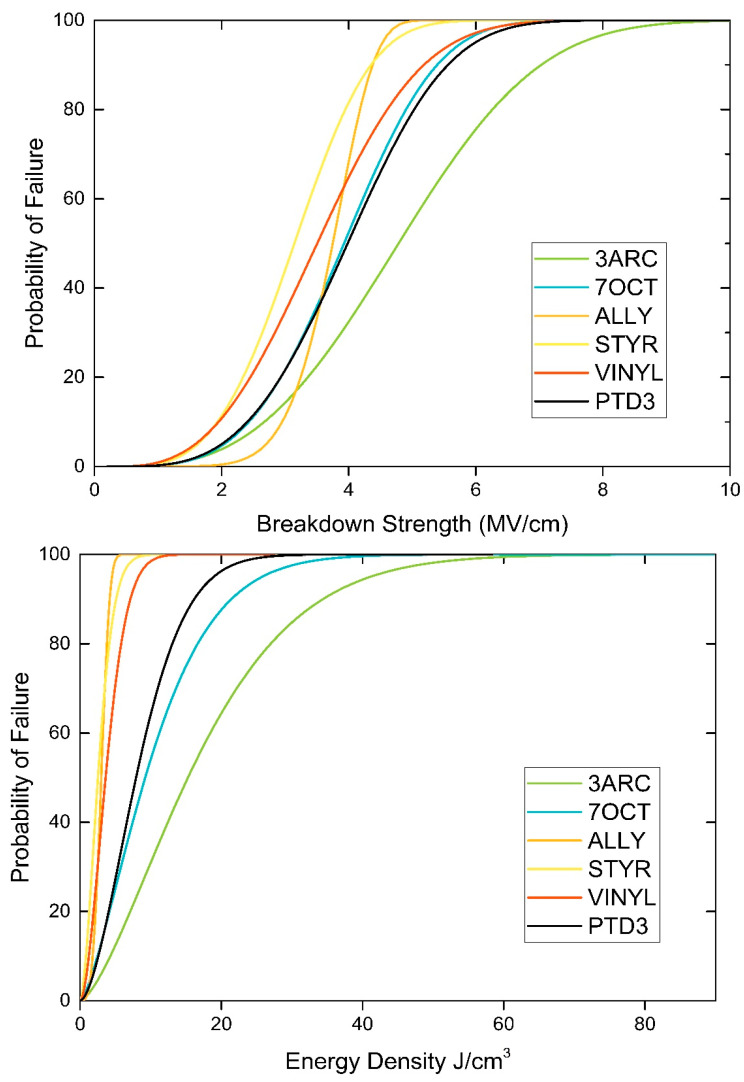
Breakdown strength and energy density at 1 kHz for the different polymers.

From the breakdown data in Figure 5, we can see that the 3ARC surface functionalization has the highest breakdown. The scale (*α*) and shape (*β*) parameters used in the Weibull analysis to create Figure 5 are shown in Table 2. This is in contrast to what was expected from our DFT predictions. The 7OCT is the second-highest breakdown, which is what our model had predicted to be the highest. The remainder of the surface functionalizations had a similar order in terms of breakdown magnitude, as predicted by our model. The film with the highest energy density, 3ARC, had a high energy density as compared with pumped hydropower and compressed air storage [29], 19.39 J/cm^3^ for 3ARC compared to 21.6 J/cm^3^ for compressed air and 7.2 J/cm^3^ for pumped hydropower. With high energy storage (combined with other factors such as lower maintenance costs), our composite is clearly suitable for grid-scale energy storage.

In examining why the order of breakdown magnitude of surface functional groups is different, we have to examine the assumptions of our original model, the results of which are summarized in the column “Calculated Electric Field Drop” in Table 2. Our model assumed a uniform polymer host around the functionalization and a certain number of surface functionalizations per BaTiO_3_ lattice sites. For the first assumption, we did not model the effects the functional groups might have on the matrix, and as discussed above, the interface between the two can change the overall breakdown of the composite by polarizing the matrix. For the second point, we can see in Table 2 that the surface functionalizations are clearly different in molecular mass, which will also affect how easy it is for the functional groups to coat the surface of the nanoparticle. Thus, to relate our model to the experiment, we must account for the coating densities of each surface functionalization.

In our previous work, we hypothesized that the functional group that disrupts the polymer matrix the least, that is, has the lowest total drop in electrical field, should have the highest overall breakdown. Our logic was that the change in overall breakdown would be proportional to how much the nanoparticle–polymer interface disrupts the electric field of the composite. This change in field gives energy to electrons that create currents and ultimately cause breakdown. As discussed above, to compare surface functionalizations more accurately would require us to look at the number of sites in each composite and use the electrical field disruption per site since there are more sites proportionally with the smaller surface functionalizations. Table 3 shows the integrated field drop for each surface functionalization calculated previously and the calculated coating density for each surface functionalization. We can see from Table 3 that different surface functionalizations have different coating densities, which are not solely proportional to the molecular weight. We also calculate the field drop per surface site [30] and extend it to calculate the field drop per unit area. This gives a more correct prediction of breakdown. The 3ARC has the lowest field drop per unit area and also the highest breakdown strength.

The other surface functionalizations remain in the same order as previously predicted. The sole incongruity is that STYR was predicted to be the 3rd highest breakdown film, but it is the 5th. This could be due to polarization effects, as it has a highly polarizable aromatic ring. We can see that the correlation does match for functionalizations that exceed or match the breakdown of neat PTD3. We can assume that the electrical behavior of those composites, which do not have as high a breakdown strength as the neat, have other, more complicated phenomena occurring, such as with STYR discussed above. We can say that our model is a success in describing useable composites and that other criteria must be used before our model can screen out undesirable, low-breakdown composites.

Although our method works for ranking most surface functionalizations, we must still look at other reasons for breakdown amongst different surface functionalizations. Previous works have hypothesized that higher ligands per nm^2^ would yield higher breakdown, as more attachment sites would yield higher total covalent bonding. This cannot be proven here without further study because although ALLY has the highest density of ligands per nm^2^, it has a middling performance in breakdown. This could be attributed to many of the molecules not adhering directly to the surface but instead being intertwined in the attached surface functionalizations. This could interfere with the ability of the ALLY molecules to crosslink with the polymer host.

An explanation that follows the data is that long repeating units increase breakdown strength. This is substantiated by looking at the two different surface functionalizations that did the best, 3ARC and 7OCT, as opposed to STYR, whose molar mass seems to derive from the aromatic ring. However, the three surface functionalizations with the lowest breakdown have similar lengths despite having markedly different performances. Perhaps there is a minimum length that provides the higher crosslinking, which would explain how these two longer functionalizations performed better than others.

To more accurately predict breakdown in the future, we need to more accurately model surface functionalizations. In our laboratory-fabricated films, we have the disadvantage of irregular surface adhesion of the surface functionalization to the nanoparticle. Steric effects likely affected the ability of the nanoparticles to crosslink in a way similar to our simulation on the surface, changing the overall electronic characteristics. Additionally, during film fabrication, xenon light irradiation could affect the surface functionalizations themselves instead of only activating the bond between the surface and polymer host. Imaging the surface of nanoparticles present in finished films using cross-sectional TEM could give a precise indication of the configuration of the nanoparticle surfaces and allow us to make more accurate computational models. Using this technique would likely lead to the need for an ensemble of surface models since there would probably be many different surface configurations. In a different approach, very tightly controlling surface functionalizations to match computational results would allow us to more easily compare experiments.

We can clearly see that more work is needed if we want to effectively model the nanoparticle–host interface and create even higher energy density capacitors. A computationally created three-dimensional electric field graph could likely provide more detail and allow us to tease out specific details related to what is going on in our composites, specifically allowing us to explain why some composites have a lower breakdown strength as compared to the neat polymer. Using this information, we can more accurately incorporate experiment-to-model functionalizations in a way that creates nanoparticle surfaces more similar to the real world. Using our method, we have a starting point for prescreening the many other thiol-ene surface functionalizations, most of which are much more expensive.

Additionally, future industrial refinement will be needed, with future work focused on creating tape-cast films on a large scale. Although industrial production is compatible with the current deposition process, a higher throughput deposition method will be needed to reach the scale required for industrial applications.

## 3. Methods

To create a thin film composite, a polymer precursor solution must be mixed with nanoparticles and crosslinked. Precursor polymer ink was made from three constituent monomers, as previously described [31]. This ink was chosen for its ease in creating films by a variety of methods, making the process easily scalable to manufacturing. These were 1,3 Diisopropenylbenzene, 2,4,6-triallyloxy 1,3,5-triazine, and Pentaerythritol tetrakis (3-mercaptopropionate), and their molecular weights were 83%, 11%, and 6%, respectively. The ink, called PTD3, was sonicated for 10 min. These monomers were chosen for their ability to crosslink by UV light. The ink itself demonstrated the highest energy storage of previously studied inkjet ink. Inkjet processing is expected in future applications of this work.

For the nanoparticles to be capable of crosslinking with the polymer, they must first be functionalized. A total of 100 nm cubic BaTiO_3_ nanoparticles (US Research Nanomaterials, Houston, TX, USA) were functionalized under nitrogen with OH groups by mixing 400 mL of hydrogen peroxide per gram of nanoparticles for four hours [32]. Then, 80 mg of these nanoparticles were combined with 500 mg of surface functionalization ((3-acryloxypropyl) trimethoxysilane, 7-octenyltrimethoxysilane, allyltrimethoxysilane, vinyltriethoxysilane, styrylethyltrimethoxysilane) and stirred under nitrogen for 12 h. Composite inks were made by mixing one volume percent functionalized nanoparticles (six weight percent) with PTD3 and sonicating for 10 min, forming a milky suspension. Their adherence of the functional groups to the nanoparticle surfaces was confirmed using Fourier Transform Infrared Spectroscopy (FTIR).

Thermogravimetric Analysis (TGA) was completed on a Thermo Scientific (Waltham, MA, USA) Q500 in order to study the density of surface functionalizations on the nanoparticle surfaces. All data were collected in a tared 100 μL platinum sample pan. The sampling protocol consisted of ramping from 25 °C to 110 °C at a rate of 10 °C min^−1^ under an inert nitrogen atmosphere (10 mL min^−1^) with data collection turned off, followed by an isothermal hold at 110 °C for 10 min to remove all moisture presence. Following the isothermal hold, data collection was turned on, and the temperature was ramped from 110 °C to 810 °C at a rate of 5 °C min^−1^ under an inert nitrogen atmosphere (10 mL min^−1^). The temperature was then held at 810 °C isothermally for 5 min. Measurements are presented in the form of percent (%) weight loss over a range of temperatures (°C).

Approximately 25 mm square, 30 µm thick copper foil (Oak-Mitsui, Hoosick, NY, USA) squares were laminated onto glass substrates to increase rigidity for spin coating. Foils were chosen because of their lower surface roughness percentage as compared to more rigid copper blanks. Spin coating was used to create films. Foils spinning in a spin coater (Laurell, North Wales, MA, USA) were sprayed with acetone using the same spin parameters as the deposition to lower the surface energy in order to increase ink adhesion. Then, 700 microliters of ink were dynamically dispensed using the first stage of an auto pipette. The spin parameters for deposition were 500 rpm for 20 s with 500 rpm/s acceleration for the dynamic dispense step and 1000 rpm for 30 s and 500 rpm/s acceleration for the spin-off step. Lower spin speeds were chosen to prevent shear thinning, which had been observed previously to result in uneven coating.

Films were cured using PulseForge curing [33], a xenon flash lamp system with computer control over pulse shape, width, and micropulses; those parameters allowed for finer control over film quality, specifically, reduced film wrinkling and edge burning. The parameters used were 470 V, 3000-microsecond pulse width, 5 micropulses, 50% duty cycle, and 1 Hz [34]. An integrated bolometer measured the output energy at 4.2 J/cm^2^. To provide the same fluence, the pulse width and the voltage were the primary parameters adjusted. Applying a higher voltage to the lamp provides a higher percentage of the outputted light being UV, which is important for our UV-sensitive inks. Additionally, the higher UV content allows us to eschew photoinitiators, allowing more material for energy storage. Films were cured in 10-pulse increments, with 30 pulses being sufficient to cure all films. Testing of cured films was performed electrically with a multimeter to test if an insulator had formed. A secondary test was to wipe the film with acetone to determine if it had cured into a solid layer.

A grid of 16 circular silver electrodes, each with 0.9 mm diameter and 500 nm thickness, was DC sputter coated through a mask onto each sample film’s surface for electrical testing. Dielectric testing was carried out using a Keysight (Santa Rosa, CA, USA) E4990A impedance analyzer, sampling 4–6 points per sample and averaging. Breakdown measurements were made using Radiant Technologies’ (Albuquerque, NM, USA) Premier II Ferroelectric tester with a 10 kV amplifier (Trek Inc., Lockport, NY, USA). Breakdown strength was determined by creating a testing program utilizing the leakage current measurement function with a 500 ms soak time and 1000 ms read time for each voltage. The voltage was increased by 20 V in each step. Dielectric failure was considered to have occurred when a leakage current of greater than 1 mA was measured at a specified voltage. Breakdown measurements were conducted 12 times in order to perform Weibull statistical analysis.

A Weibull plot was constructed for each ink from at least 8 measurements. Each data set was fit with a linear trend line to calculate the shape and scale parameter. The shape parameter *β*, which describes the statistical variation of the breakdown voltage, is taken from the slope of the trend line. The scale parameter *α*, the field at which there is a 63% chance of failure, is calculated by taking the exponential of the y-intercept over the slope. Once the scale and shape parameters are defined, a Weibull distribution that shows the failure probability for all possible fields can be constructed using Equation (2).

(2) $$P\left(E_{B}\right)=1-e^{\left(-\left(\frac{E_{B}}{\alpha \;}\right)\beta \;\right)}\;$$

## 4. Conclusions

The previous model was refined by including feedback from experimental results. The refined model is expected to be an effective tool to prescreen surface functionalizations and identify surface functionalizations with higher breakdown fields. However, further refinement might be needed. We have seen that dielectric polarization may play an important role in determining how some surface functionalizations change breakdown properties. We also identified that 3ARC surface functionalization is more effective in increasing the breakdown voltage of composites prepared with click chemistry. As these films were made with scalable techniques, they can be useful in grid-scale storage. Our method was aimed at relating breakdown to first principles calculation, and we were able to show that our method of modeling electric field drop predicted the most promising candidates successfully. The experimental feedback from this method showed that more experimental control is needed to effectively look at atomic-scale energy fields and how they relate to breakdown. We also demonstrated how our method could be used to screen potential candidates using this method, saving time and money in the process, and creating even higher energy density materials by using doped nanoparticles.

## Figures and Tables

**Table 1:** Abbreviations and full names of surface functionalizations studied.

Abbreviation	Full Name
3ARC	(3-acryloxypropyl)trimethoxysilane
7OCT	7-octenyltrimethoxysilane
ALLY	Allyltrimethoxysilane
VINYL	Vinyltriethoxysilane
STYR	Styrylethyltrimethoxysilane

**Table 2:** Weibull Analysis Parameters.

Film Name	*α* (Breakdown Voltage [MV/cm])	*α* (Energy Density [J/cm^3^])	Energy Density [Whr/kg]	*β*	Permittivity (at 1 kHz)
PTD3 (No NPs)	4.10	8.80	2.22	3.37	11.90
3ARC	5.07	19.39	4.89	2.96	17.14
7OCT	3.64	3.61	0.91	2.85	6.20
ALLYL	3.63	3.30	0.83	7.19	5.69
STYR	3.17	3.10	0.78	3.40	7.02
VINYL	4.02	14.42	3.64	3.54	20.27

**Table 3:** Calculated surface density of functionalizations and electric field drop per nm^2^.

Surface Functionalization	TGA Weight Loss Percentage	Molar Mass (g/mol)	Ligands/ Particle	Ligands/nm^2^	Calculated Electric Field Drop % (per Site)	Local Field Drop %/nm^2^
3ARC	5.88	304.37	7.00 × 10^5^	23	2.9199	68
7OCT	4.71	232.39	7.34 × 10^5^	24	2.9048	71
ALLY	5.51	162.26	1.23 × 10^6^	41	3.0349	124
STYR	5.64	252.38	8.09 × 10^5^	27	2.9304	79
VINYL	5.52	190.31	1.05 × 10^6^	35	3.0248	106

## Data Availability

The data presented in this study are available on request from the corresponding author.
